# A hybrid recommender system based on data enrichment on the ontology modelling

**DOI:** 10.12688/f1000research.73060.1

**Published:** 2021-09-17

**Authors:** Lit-Jie Chew, Su-Cheng Haw, Samini Subramaniam

**Affiliations:** 1 Faculty of Computing & Informatics, Multimedia University, Cyberjaya, Selangor, 63100, Malaysia; 2 AirAsia Berhad, KLIA, Selangor, 64000, Malaysia

**Keywords:** Information Retrieval, Ontology, Recommender System, Collaborative Filtering, Content-based System, Hybrid Recommender System

## Abstract

**Background: **A recommender system captures the user preferences and behaviour to provide a relevant recommendation to the user. In a hybrid model-based recommender system, it requires a pre-trained data model to generate recommendations for a user. Ontology helps to represent the semantic information and relationships to model the expressivity and linkage among the data.

**Methods: **We enhanced the matrix factorization model accuracy by utilizing ontology to enrich the information of the user-item matrix by integrating the item-based and user-based collaborative filtering techniques. In particular, the combination of enriched data, which consists of semantic similarity together with rating pattern, will help to reduce the cold start problem in the model-based recommender system. When the new user or item first coming into the system, we have the user demographic or item profile that linked to our ontology. Thus, semantic similarity can be calculated during the item-based and user-based collaborating filtering process. The item-based and user-based filtering process are used to predict the unknown rating of the original matrix.

**Results:** Experimental evaluations have been carried out on the MovieLens 100k dataset to demonstrate the accuracy rate of our proposed approach as compared to the baseline method using (i) Singular Value Decomposition (SVD) and (ii) combination of item-based collaborative filtering technique with SVD. Experimental results demonstrated that our proposed method has reduced the data sparsity from 0.9542% to 0.8435%. In addition, it also indicated that our proposed method has achieved better accuracy with Root Mean Square Error (RMSE) of 0.9298, as compared to the baseline method (RMSE: 0.9642) and the existing method (RMSE: 0.9492).

**Conclusions:** Our proposed method enhanced the dataset information by integrating user-based and item-based collaborative filtering techniques. The experiment results shows that our system has reduced the data sparsity and has better accuracy as compared to baseline method and existing method.

## Introduction

A Recommender System (RS) is a system that can provide item recommendation to a user based on their personalized interest. The attention for RS has increased dramatically over the past decade in various industries and domains such as e-commerce and online video streaming. There is a crucial need for having a system that can filter the numerous data around us as we are living in the area of the Internet with humungous data transactions and exchanges daily. With a properly implemented RS, the user will get a personalized recommendation based on the preferences, interest, rating, search results, the similarity between other users and so on. There are various successful use cases where RS helps in increasing the revenue of industrial, especially on online businesses. E-commerce companies such as eBay ^1^ and Amazon ^2^ have made use of RS to promote their products to the targeted customer. On the other hand, online video streaming company such as Netflix ^3^ and YouTube ^4^ have also implemented multiple types of RS in their system.

Generally, there are two types of RS: (1) content-based filtering (CB), and (2) collaborative filtering (CF). The CB RS provides recommendations to a user by using user preferences or history while the CF RS generates the recommendations based on the relationship between the user and item. These two methods have their advantages and shortcomings. As such, to combine the advantages and eliminate the shortcoming of each specific method, a new group of RS named hybrid RS has emerged. According to a recent survey in 2020 ^5^, most of the recently proposed RS techniques fall under this group. Besides that, the most proposed hybrid RS combine at least one CF method in their system. CF method can be further classified as memory-based and model-based CF. A memory-based CF suggest item based on the similarity between user or item while the model-based CF builds the model by learning the interaction between user and item. There are a few researchers who focus on enhancing the model performance by fine-tuning the parameter and method in the model development process. However, the accuracy of the model built depends on the quality of the data ^6^.

On the other hand, ontology helps to structure the data in a way that the entities are connected within the database ^7^. Thus, the relationship between each entity is preserved. Semantic similarity can be easily calculated by various method that the ordinary method may not be able to discover. Ontology has been proven to help in increasing the accuracy of the RS and decrease the cold start issues ^8, 9^. With ontology, Manuela *et al.* ^10^ reduced the fake neighbours’ problem cause by the CF method. Tarus *et al.* ^11^ proposed an E-learning RS based on RS and the accuracy is better than using only CF without ontology. Shaikh *et al.* ^12^ proposed an ontology-based RS in an e-commerce website. User behaviour on the website has been captured as implicit feedback to the RS. Gohari and Tarokh ^13^ proposed a hybrid method that using ontology to structure the data. User-based (UB) CF and item-based (IB) CF were used to generate the recommendation. Bagherifard *et al.* ^14^ proposed a hybrid approach that utilizing ontology in CB and CF hybrid RS. In their approach, the user has been clustered before calculating pass to CB and CF. This reduces the compute time of the CF RS. Celyan *et al.* ^15^ proposed SEMCBCF, which is an ontology hybrid RS that extended from CBCF ^16^, which is a CF RS without ontology integrated. The semantic similarity between items was calculated in their proposed system. The weighted average algorithm was used to combine the different similarity value. Nilashi *et al.* ^17^ proposed an ontology hybrid recommendation that using IB and UB CF together with the clustering method to reduce overgeneralization. On another separate research, Liu and Li ^18^ proposed an ontology CF RS based on Singular Value Decomposition (SVD). By employing the ontology as the data representation, the data sparsity has been decreased and the empty value of the user-item matrix was filled up based on IB CF. Inspired by their work, we proposed to address on enriching the data representation by means of ontology enrichment to give a more accurate recommendation. The summary of the recent publications has been done in Table 1.

**Table 1.** Recommendation system type and advantages of each publication.

Publication	RS Type	Advantages
**10**	CF	Reduce fake neighborhoods’ problem.
**11, 12**	CF	Able to capture implicit feedback.
**14**	Hybrid (CB and CF)	User has been clustered in ontology to reduce compute time.
**13, 15, 17**	Hybrid (IB and UB CF)	13 User demographic is used. 15 Unknown rating predicted by CB before CF process. 17 Clustering item and user to reduce overgeneralization.
**18**	Hybrid (IB and model-based CF)	Enriching the matrix by IB CF before the model-based CF process.

In our proposed method, we focus on how to enrich the data information with ontology in order to increase the accuracy of the model-based RS. We proposed a method to enrich the user-item rating matrix by using the semantic similarity calculated from ontology. We added a UB RS to the item-based RS to generate the predicted rating that used to fill the user-item matrix to improve on the accuracy. In addition, our proposed approach will also reduce the main problem that usually faced in the model training, which is the data sparsity issue. With the predicted rating filled in the original user-item matrix, it can fill up the unknown value thus reduce the sparsity and increase the model training result. The experiment evaluations demonstrated that we have achieved higher accuracy and decrease the data sparsity problem of the original matrix.

## Methods

**Figure 1.**
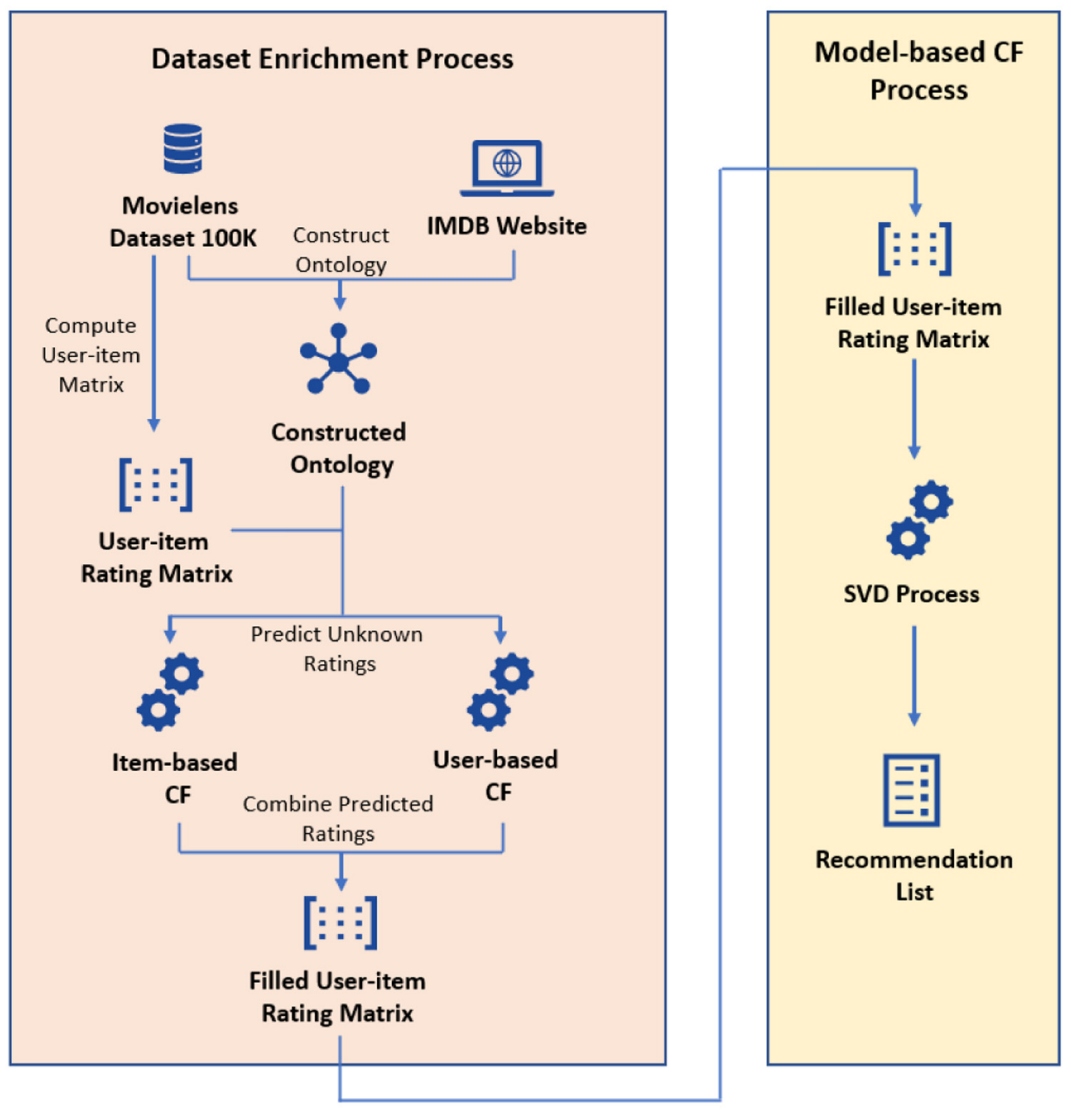
Flow diagram of the proposed method.

Insipred by the data enrichment method proposed by Liu and Li ^18^, we extended the work and proposed a hybrid method that used ontology to model the data. The semantic similarity between each attribute will be calculated by using the ontology structure. The semantic similarity will be used in the rating prediction in IB CF and UB CF. The flow diagram of our proposed method is illustrated in Figure 1. The proposed method consists of four parts:

1. Crawling extra movie information from IMDB and construct the ontology

2. Unknown Rating prediction by IB and UB CF

3. Combine predicted ratings and forms a filled user-item rating matrix

4. Model-based CF.

**Figure 2.**
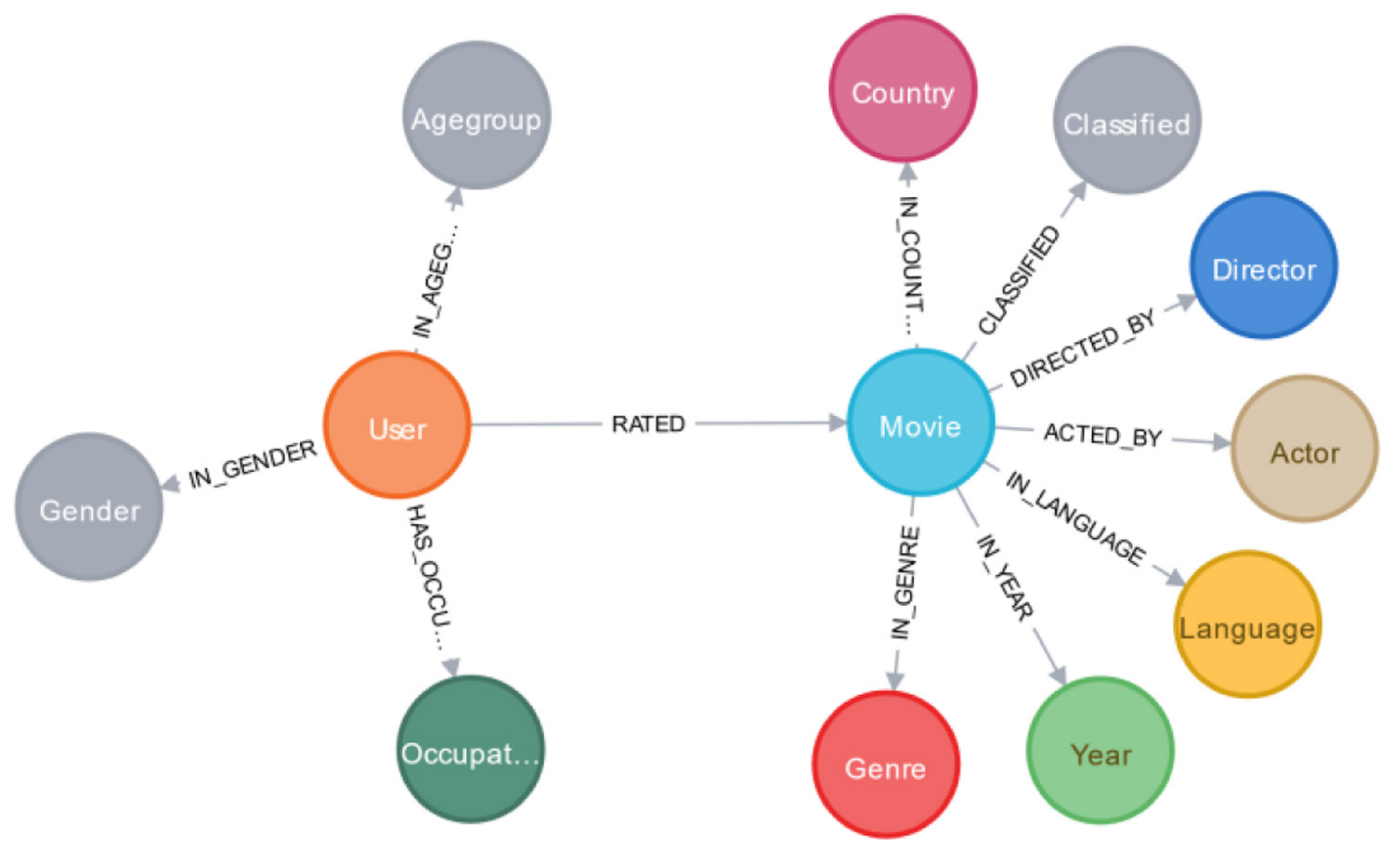
Ontology constructed based on the MovieLens dataset.

We have selected the MovieLens 100K dataset as this is the standard dataset used for benchmarking purpose. This dataset contains 100K rating records with 1682 movie data and 943 user profile details. However, the movie information of the MovieLens dataset is limited. To have more details for the movie, we crawled the extra information from the IMDB website such as movie country, classified, director, actors, and so on. After all the data had been crawled, we constructed the ontology representation for the dataset (see Figure 2). In the ontology representation, all the attributes nodes were connected with each other via the relationship edges. The two main nodes were User and Movie connected through their related profile node. 

The semantic similarity of the dataset can be easily counted from the ontology constructed above. We used the IB CF and UB CF together to predict the unknown value from the original user-item matrix. The IB CF calculate the semantic similarity by considering the relationships between items. We used the Jaccard similarity index in calculating semantic similarity. Jaccard similarity measures the similarity by taking the percentage of the intersection of two sets of data. The formula is depicted in Equation (1).


$$J(A,B)\;=\;\frac{\left|A\cap B\right|}{\left|A\cup B\right|}\;=\;\frac{\left|A\cap B\right|}{\left|A\right|+\left|B\right|-\left|A\cap B\right|}\;(1)$$

Where J(A, B): the Jaccard similarity index between data A and data B.

From the process above, we got the movie-movie similarity by each feature of the movie (see Table 2). We then combined all the movie-movie similarity by a weighted average algorithm, where the weight variables were decided by experiment evaluation to get the best combination.

**Table 2.** An example of a movie-movie similarity matrix.

	Movie1	Movie2	Movie3	Movie4	…
**Movie1**	1	0.35	0.86	0.5	…
**Movie2**		1	0.2	0.6	…
**Movie3**			1	0.88	…
**Movie4**				1	…
**…**					1

After completing the IB similarity calculation, we were able to predict the unknown rating values in the user-item matrix. The theory of the prediction is finding the related movie ratings rated by the specific user. The formula used is shown in Equation (2).


$$Predicted_{u,m}\;=\;\frac{\sum FinalSim_{i}\times a_{u,i}}{\sum FinalSim_{i}}\;(2)$$

Where i: the movie rated by the user, a: rating, u: user, m: movie

The algorithm first took all the movie rated by the specific user and compare to the similarity calculated. It then summed up the predicted value by using the weighted average method where the weight is the similarity of the movie to that specific movie. The predicted value put in a temporary matrix which was later combined with UB RS.

In the UB CF, we applied similar methods from the IB CF above. First, we calculated the similarity of each user features then combined it with a weighted average algorithm. With the user-user similarity calculated, we then predicted the empty movie rating by finding similar users. The similar users’ rating to that specific movie was combined by the weight algorithm.

Once the two IB CF and UB CF methods were completed, the two predicted rating were then combined by using the weighted average algorithm to get the final predicted rating for filling the empty original user-item matrix. After the filling process was completed, it was then passed to the model-based CF to construct the model. The CF model used in this paper was SVD. SVD decomposed the matrix into two lower dimensionality matrix and extracted the latent features. It is a famous method used in the model-based CF.

## Results

In the evaluation, we have compared the result from the baseline model that based on SVD method alone to predict rating and an existing method that uses the IB CF to enrich the original user-item matrix. 

The proposed system was developed using Jupyter Notebook 6.4.0 in Python 3.6 and Linux environment (Ubuntu 18.04). The Neo4j database has been used to store the data as the it is a graph database that our ontology representation will maintain in the data model. We applied the Root Mean square error (RMSE) algorithm to determine the accuracy of the system. It is a common approach to determine the predictive accuracy of the model ^19^. It gives a relatively high weight to large errors. The smaller the RMSE value, the more accurate the model is.

**Figure 3.**
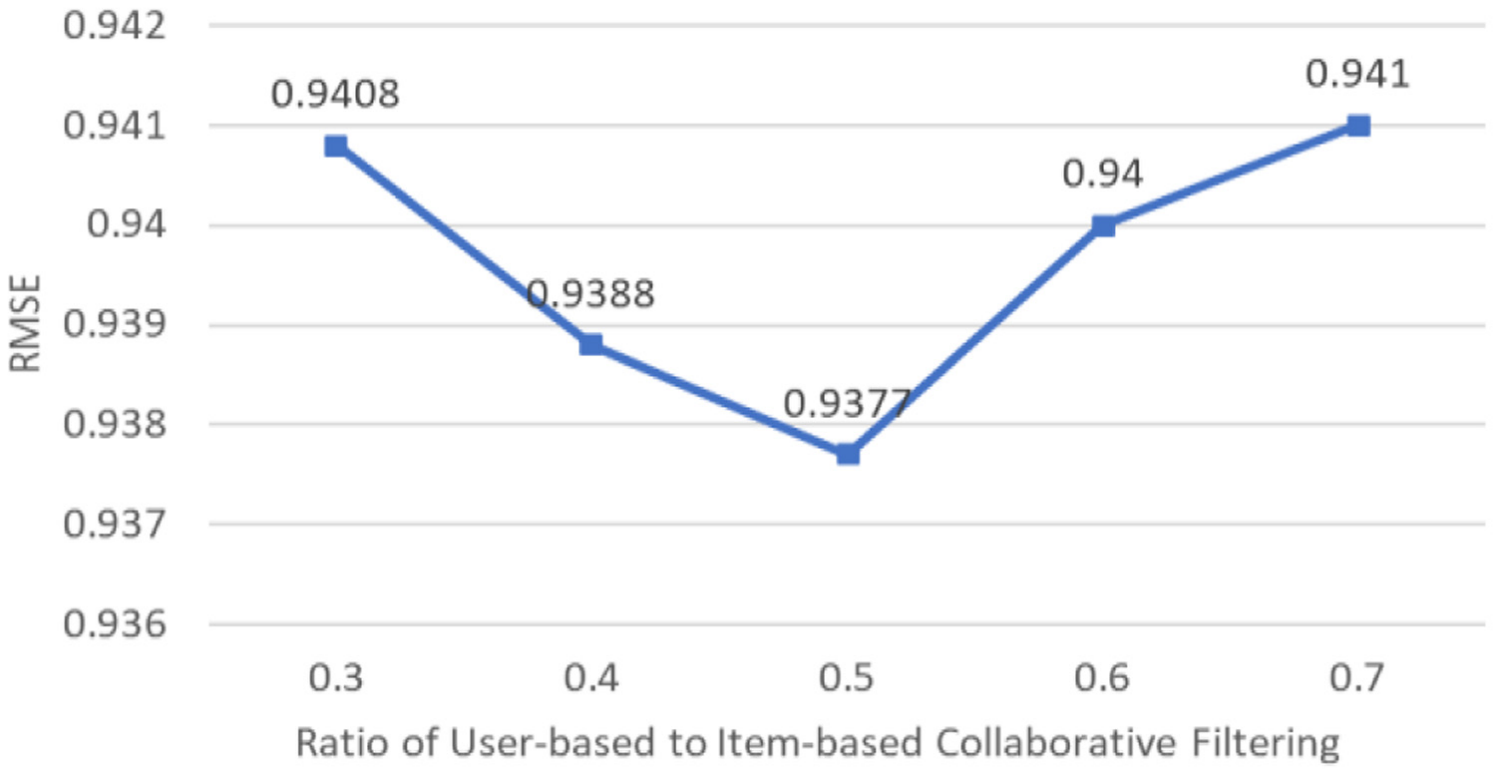
RMSE of Various Ratios of User-based to Item-based Collaborative Filtering.

Several experiments have been done to decide the weight variable used in combining the IB CF and UB CF. Various weightage variables ranging from 0.3 to 0.7 have been tested. Figure 3 shows that the best accuracy is achieved with a weightage of 0.5.

**Figure 4.**
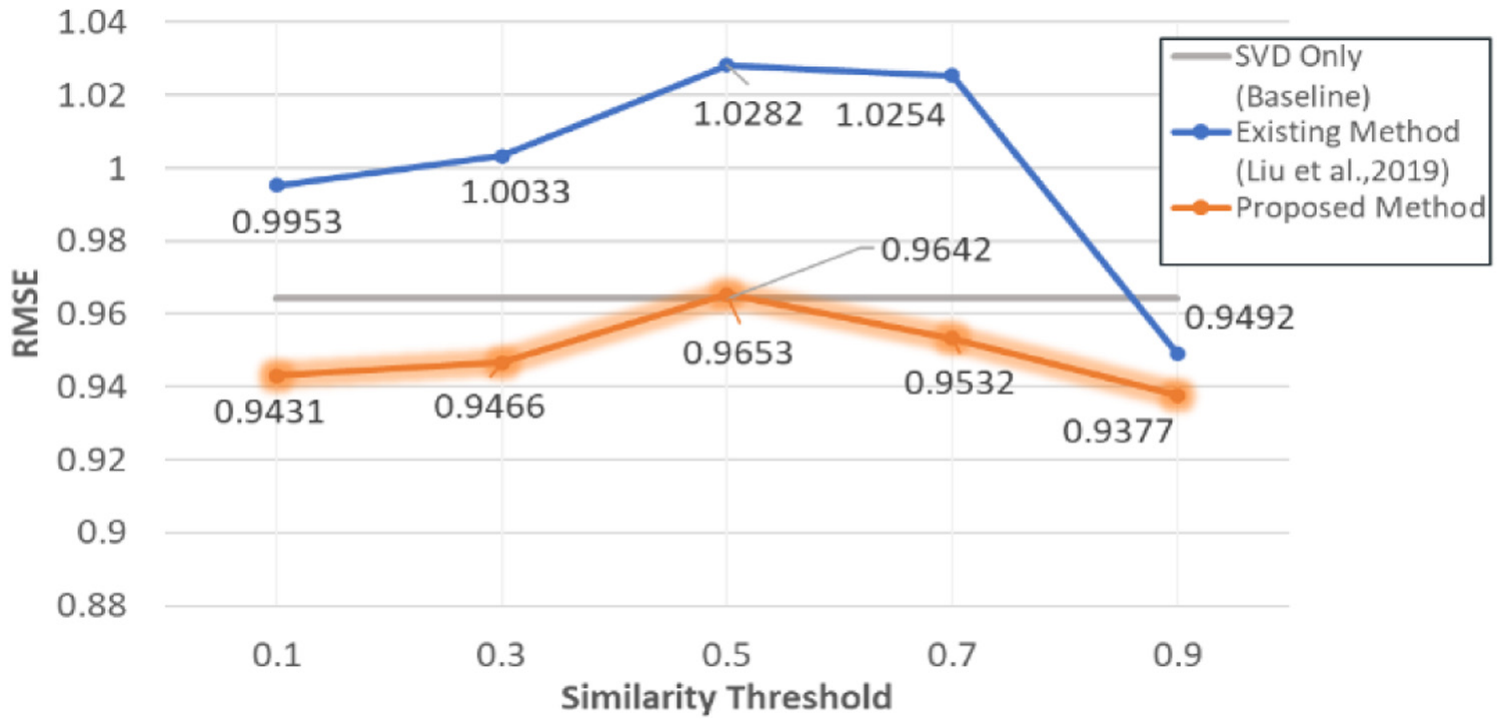
RMSE comparison with different similarity threshold and methods.

**Figure 5.**
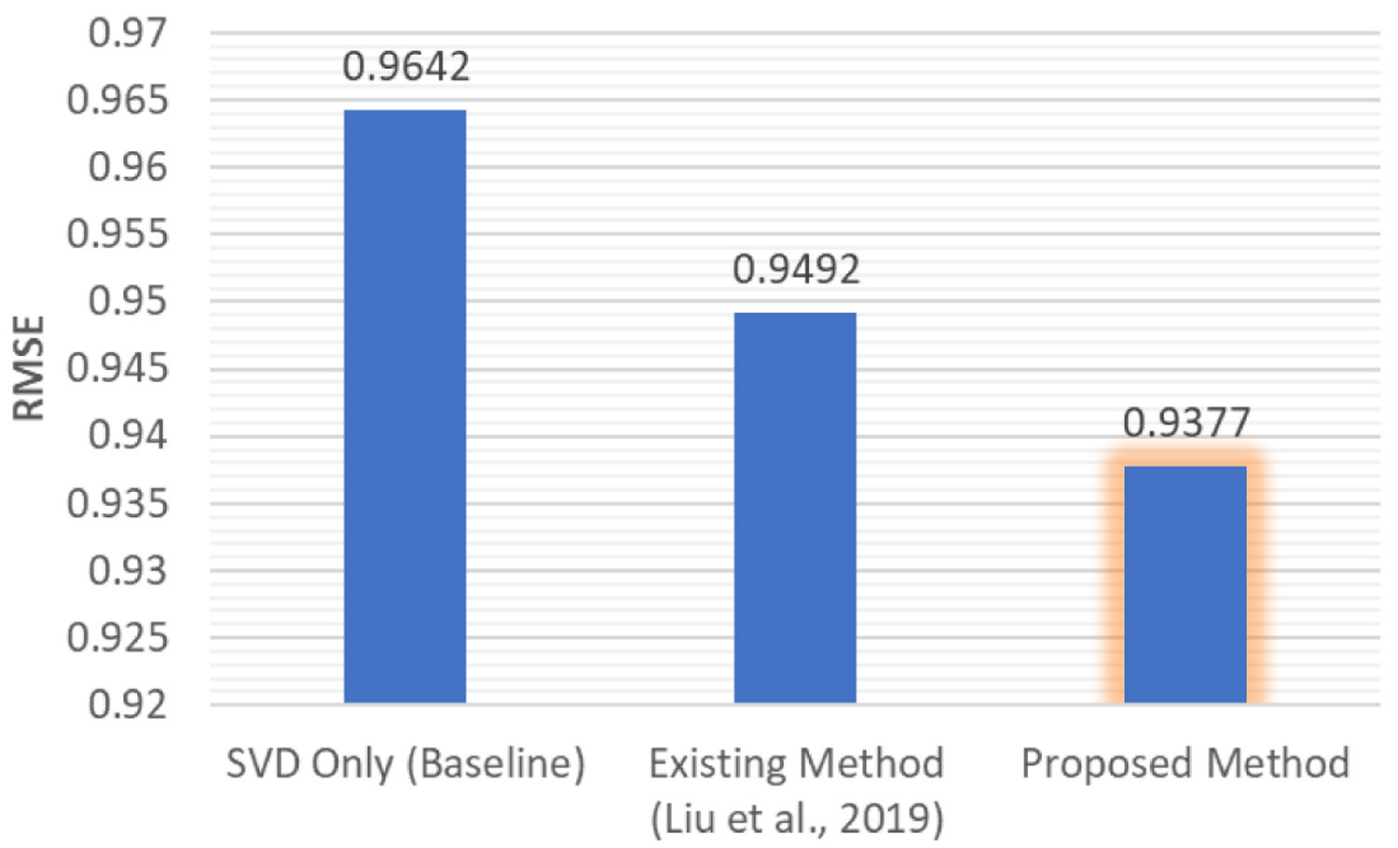
Lowest RMSE value comparison between various methods.

A similarity threshold was applied in the system to prevent destroying the original information of the original matrix when filling the empty value. Figure 4 shows that the accuracy of the model was affected by the similarity threshold. Overall, our proposed method had the lowest RMSE value across the similarity threshold testing (see Figure 5).

The experiment evaluations indicated that our proposed approach had the lowest RMSE value. With the unknown rating filled by IB and UB CF before passing to the model-based CF, the data sparsity also decreased from 0.9542% to 0.8435%.

## Discussion

**Figure 6.**
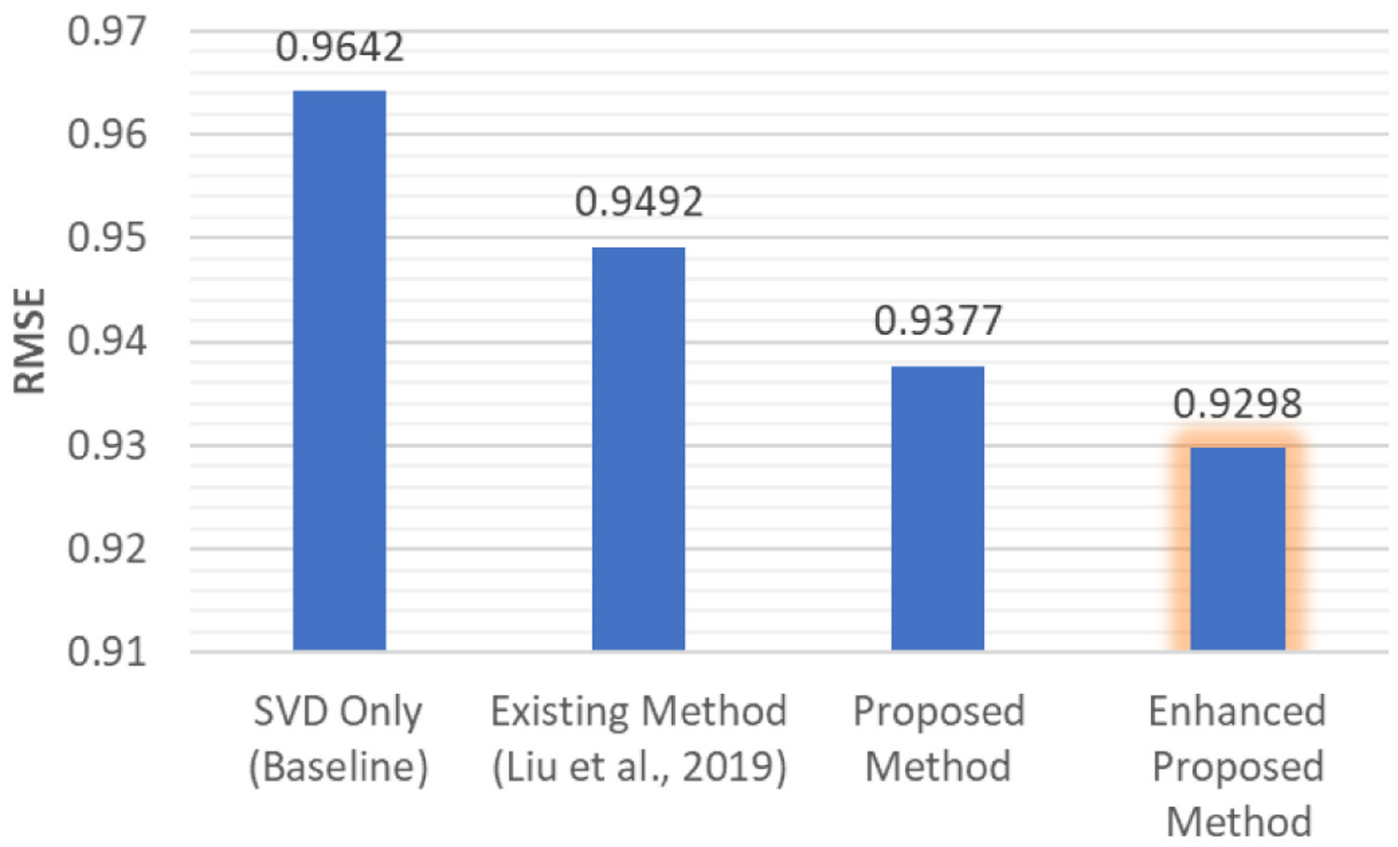
Lowest RMSE value comparison between various methods.

From the experimental results in the earlier section, we observed that adding the IB CF method to enrich the original data helped to increase the accuracy of the model-based CF RS. It helped to boost the information of the original matrix while not destroying the original information. The added user-item CF method allowed the system to get more accurate similar user and items. However, we still want to know if our proposed method works in other model-based CF RS. Hence, we change the SVD model to the SVD++ model as the enhanced proposed method and re-run the experiment. SVD++ is an extended work from SVD, which achieve better accuracy by optimizing the algorithm to consider implicit feedback ^20^. In our experiments, the results in Figure 6 below show that the enhanced ptoposed method with SVD++ outperforms any other method we used above with the enriched data. This helps to verify that our method can be applied to not only SVD, but any other model-based CF RS. 

From all the results above, it shows that our proposed method can increase the accuracy of the model-based CF RS. By adding the UB CF method to the existing method proposed by Liu and Li ^18^ that employed only the IB CF method, we can achieve better accuracy than the existing method. This is due to the added UB CF method which allows the system to find the related item by user demography, whereas the IB CF method is not able to do it. 

## Conclusions

In this paper, we reviewed the current ontology based RS and proposed a data enrichment method which uses ontology in a hybrid RS. The proposed method increases the model-based CF RS input data quality by adding the UB CF to the existing IB CF method. Both methods use the structure of ontology to calculate the semantic similarity and, subsequently, fill the unknown rating values of the original user rating matrix. Experiment results indicated that the data sparsity problem has been minimized and the accuracy of the RS system has been increased.

Several improvements can be conducted in future including algorithm optimization. The current offline model building algorithm takes time to process and can be optimized as parallel processing to improve the processing time. Besides, the semantic similarity calculation can be changed to the level-based calculation to fully utilise the benefits of having ontology in the system.

## Data and Source Code Availability

### Underlying data

Zenodo: chewljie/dataset-enrichment-RS: V1.0 Initial Release, https://doi.org/10.5281/zenodo.5418122

This project contains the following underlying data:

• MovieLens 100K. ( https://grouplens.org/datasets/movielens/100k/)
• Extra movie details from OMDb API.
  ( https://www.omdbapi.com/)

Data are available under the terms of the Creative Commons Attribution 4.0 International license (CC-BY 4.0).
