# Supplementary material for: A hybrid recommender system based on data enrichment on the ontology modelling
Source: F1000Res. 2021 Sep 17;10:937. [Version 1] doi: 10.12688/f1000research.73060.1 (PMC8609391; doi:10.12688/f1000research.73060.1)
Supplement: Peer review history

# Peer review: Reviewer response for version 1

**DOI:** 10.5256/f1000research.76683.r97017

**Published:** 2021-11-22

**Authors:** Heru Agus Santoso

**Affiliations:** 1 Faculty of Computer Science, Dian Nuswantoro University, Semarang, Indonesia

The article has a good chance of being accepted, but  requires explanation in detail:

1. Please check new references, there are other types of recommended systems such as knowledge-based, hybrid etc.
2. The study focuses on enriching information of data using ontology, explain:
  a. Method/technique how to crawl and construct the ontology;
  b. Technique to predict rating using IB and UB;
  c. How to combine predicted rating and user-item rating form. 
3. Explain clearly, the method to improve accuracy by carrying out semantic similarity using ontology. 
4.  RMSE is not algorithm (see the Result), is there relation with the use of ontology? If so, how can it improve the performance of RS?

Is the work clearly and accurately presented and does it cite the current literature?

Partly

If applicable, is the statistical analysis and its interpretation appropriate?

Yes

Are all the source data underlying the results available to ensure full reproducibility?

Yes

Is the study design appropriate and is the work technically sound?

Partly

Are the conclusions drawn adequately supported by the results?

Partly

Are sufficient details of methods and analysis provided to allow replication by others?

Partly

Reviewer Expertise:

Information retrieval, ontology, machine learning

I confirm that I have read this submission and believe that I have an appropriate level of expertise to confirm that it is of an acceptable scientific standard.

# Peer review: Reviewer response for version 1

**DOI:** 10.5256/f1000research.76683.r94868

**Published:** 2021-10-21

**Authors:** Dana Sulistyo Kusumo

**Affiliations:** 1 School of Computing, Telkom University, Bandung, Indonesia

This article proposes data enhancement of hybrid recommender system using ontology to model user and item relationship. This can address the cold start problem of the recommender system because it can provide information when a new user or item first comes to the recommender system. This work extends Liu and Li1 that employed only the item-based collaborative filtering method. However, the writing of this paper can be improved in the following aspects. The authors can add equations and formulas for steps in the Methods section, so that others can easily evaluate and replicate this work. As the focus of this paper is ontology extension from the previous research, please add a detailed explanation about the function of data enrichment and the ontology modeling in the Methods section. I suggest to explain how the ontology structure can be used in different cases and the scope of complexity of the ontology structure. In addition, please add more discussion about the data enrichment and use of ontology in the Discussion section.

Is the work clearly and accurately presented and does it cite the current literature?

Yes

If applicable, is the statistical analysis and its interpretation appropriate?

I cannot comment. A qualified statistician is required.

Are all the source data underlying the results available to ensure full reproducibility?

Yes

Is the study design appropriate and is the work technically sound?

Yes

Are the conclusions drawn adequately supported by the results?

Yes

Are sufficient details of methods and analysis provided to allow replication by others?

Partly

Reviewer Expertise:

Recommender system, information architecture, software engineering

I confirm that I have read this submission and believe that I have an appropriate level of expertise to confirm that it is of an acceptable scientific standard.

# Peer review: Reviewer response for version 1

**DOI:** 10.5256/f1000research.76683.r94869

**Published:** 2021-10-04

**Authors:** Rajkumar Kannan

**Affiliations:** 1 Bishop Heber College, Tiruchirappalli, Tamil Nadu, India

Author Conclusion

 In this paper, the authors reviewed the current ontology based RS and proposed a data enrichment method which uses ontology in a hybrid RS. The proposed method claims to increase the model-based CF RS input data quality by adding the UB CF to the existing IB CF method. Both methods use the structure of ontology to calculate the semantic similarity and, subsequently, fill the unknown rating values of the original user rating matrix. Experiment results indicated that the data sparsity problem has been minimized and the accuracy of the RS system has been increased.

 Reviewer comment

 Details, such as, number of objects created, relationships, download link of the ontology, that is created and leveraged are not presented and justified. Comparison with just one dated approach is not adequate either. Hence, the paper is not recommended for indexing at this present stage.

Is the work clearly and accurately presented and does it cite the current literature?

Partly

If applicable, is the statistical analysis and its interpretation appropriate?

No

Are all the source data underlying the results available to ensure full reproducibility?

Yes

Is the study design appropriate and is the work technically sound?

No

Are the conclusions drawn adequately supported by the results?

No

Are sufficient details of methods and analysis provided to allow replication by others?

No

Reviewer Expertise:

Information retrieval, Web mining, machine learning, deep learning

I confirm that I have read this submission and believe that I have an appropriate level of expertise to state that I do not consider it to be of an acceptable scientific standard, for reasons outlined above.
